# Amylenes Do Not Lead to Bacterial Mutagenicity in Contrast to Structurally Related Epoxides

**DOI:** 10.1155/2014/592434

**Published:** 2014-01-06

**Authors:** Götz A. Westphal, Carolin Tüshaus, Christian Monsé, Nina Rosenkranz, Thomas Brüning, Jürgen Bünger

**Affiliations:** IPA-Institute for Prevention and Occupational Medicine of the German Social Accident Insurance, Ruhr-Universität Bochum, 44789 Bochum, Germany

## Abstract

Amylenes are unsaturated hydrocarbons (C_5_H_10_), such as 1-pentene, 2-pentene, 2-methyl-but-1-en (3-methyl-1-butene), 2-methyl-but-2-en (isopentene), and 3-methyl-but-1-en. We investigated bacterial mutagenicity of 1-pentene, 2-pentene, and 3-methyl-but-1-en in the Ames test. 2-Pentene was investigated as racemate and as pure diastereomers. We included the methyltransferase deficient *Salmonella * Typhimurium strain YG7108 and the application of a gas-tight preincubation to reduce the risk of false negative results. 1,2-Epoxypentane which may arise from 1-pentene was used as positive control. None of the investigated amylenes showed mutagenic effects, whereas 1,2-epoxypentane was mutagenic exceeding 100 **μ**g per plate. An exceptional high reverse mutation in the negative control plates in the experiments with 1,2-epoxypentane was obviously caused by evaporation into the incubator which was shown by placing the control plates in a separate apparatus. No differences were seen upon use of YG7108 and its parent strain TA1535. In conclusion, 1,2-epoxypentane is most probably not a substrate of the deleted bacterial methyltransferases. The comparison of the bacterial mutagenicity of the investigated amylenes and 1,2-epoxipentane suggests that epoxidation of amylenes in the S9-mix does not proceed effectively or is counterbalanced by detoxifying reactions. The assessment of mutagenic effects of short chained aliphatic epoxides can be underestimated due to the evaporation of these compounds.

## 1. Introduction

**Figure 1:**
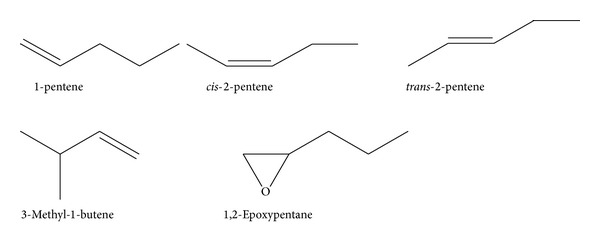


Amylenes denote a group of unsaturated hydrocarbons (C_5_H_10_), including unbranched compounds such as 1- and 2-pentene (*α*- and *β*-amylene) as well as branched compounds such as 2-methyl-but-2-en (isopentene) [CAS 513-35-9] and 3-methyl-1-butene [CAS 563-45-1] (Figure 1). Amylenes are used in the synthesis of amylphenols, isoprene, and pentanoles and can occur in gasoline or diesel fuel [1]. In addition amylenes are used as stabilizers in solvents; dichloromethane (DCM), for example, can contain up to 40 ppm.

Short chained unsaturated hydrocarbons react with biological molecules by electrophilic addition and can easily be epoxidized [2]. Both reactions can lead to mutagenic effects [3]. Theoretically, amylenes may react in a similar way. Short chained aliphatic epoxides such as ethylene oxide, 1,2-epoxypropane, and 1,2-epoxybutane are mutagenic in the bacterial reverse mutation assay [3]. Epoxidation of 1-pentene is expected to yield 1,2-epoxypentane (propyloxirane). A study which is available at ECHA reports week mutagenicity of 1,2-epoxypentane at and above 5000 *μ*g/plate [4]. Therefore, mutagenic properties of amylenes are suspected. However, available toxicological data of most amylenes are fragmentary despite their frequent use. The available data show no bacterial mutagenicity for 1-pentene [5]. We were not able to identify studies which investigated bacterial mutagenicity of* cis*- and *trans*-2-pentene and 3-methyl-1-butene.

Amylenes and their possible oxidation products show close structural relationship to known mutagens and carcinogens. However, chain length and structure have a strong impact on the mutagenicity of such short chained epoxides: a methyl group in or near the oxirane ring prevents mutagenic effects in case of the isoprene monoepoxide. Isoprene-dioxide (2-methyl-1,2,3,4-diepoxybutane) on the other hand is strong mutagenic [6]. The same is true for 2,3-epoxybutane which is a direct mutagen, whereas its 2-methyl derivative shows no mutagenicity [7].

The detection of mutagenic effects of low molecular weight compounds can be hampered by several factors such as evaporation [8, 9] and bacterial DNA repair [10]. The latter was first demonstrated using methyltransferase deficient tester strains such as YG7108 (*ogt* ^*−*^ and* ada* ^*−*^) [11]. In addition, strong stereoselective effects regarding mutagenicity were reported in case of *cis*- and *trans*-2,3-epoxybutane [12] and for the diastereoisomers of 3-bromo-1,2-epoxycyclohexane [13]. We therefore compared 1-pentene, 2-pentene,* cis*- and *trans*-2-pentene, 3-methyl-but-1-en, and 1,2-epoxypentane in the bacterial reverse mutation test.

## 2. Methods

We investigated 1-pentene [CAS 109-67-1], 2-pentene [CAS 109-68-2], *cis*- [CAS 627-20-3] and *trans*-2-pentene [CAS 646-04-8], 3-methyl-1-butene [CAS 563-45-1] (Sigma-Aldrich, Steinheim, Germany), and 1,2-epoxypentane [CAS 1003-14-1] (CHEMOS GmbH, Regenstauf, Germany) using the bacterial reverse mutation assay, with the *S.* Typhimurium strains TA98, TA100, TA1535 [14] (TA1535 only in case of 1,2-epoxypentane), and the methyltransferase deficient tester strain YG7108 *(ogt* ^*−*^ *, ada* ^*−*^) which is derived from TA1535 [11]. The test was carried out with and without metabolic activation by rat liver enzymes (S9) as well as gastight preincubation in gas-tight autosampler vials [8]. Since 3-methyl-1-butene is gaseous at room temperature it was withdrawn from a pressure cylinder and condensed at approximately –18°C using an ice/NaCl mixture. The condensate was filled in a headspace vial and transferred with a gas-tight syringe to the autosampler vials which contained the tester strains and the S9-mix or equivalent amounts of phosphate buffered saline.

We performed at least two independent experiments for every substance. The compounds were tested up to toxic concentrations but not above 5000 *μ*g/plate. Acceptance criteria for a positive test were positive and negative controls within the range of the historical controls (see Table 1) and a reproducible, dose-dependent increase of reverse mutations across at least 2 consecutive concentrations, with a maximum exceeding the base rate by at least two times [15]. Counting was performed by an automatic colony counter (BioCount, Bio-Sys GmbH, Karben, Germany).

*Chemicals and Test Compounds.* Phenobarbital/*β*-naphthoflavone induced S9 was purchased from MOLTOX, Trinova Biochem GmbH (Giessen, Germany). 1-Pentene, 2-pentene, *cis*- and *trans*-2-pentene, and 3-methyl-1-butene (95% purity, Sigma-Aldrich, Steinheim, Germany) were freshly dissolved in DMSO prior to each experiment. 1,2-Epoxypentane (98%), methyl methane sulfonate (99%), *N*-nitrosodiethylamine (99%), 2-aminofluorene (97%), and 2-aminoanthracene (95%) were purchased from Sigma-Aldrich, Steinheim, Germany. 3-Nitrobenzanthrone (99%) was purchased from Biochemical Institute for Environmental Carcinogens, Professor Dr. Gernot Grimmer-Foundation, Grosshansdorf, Germany.

Calculations of means and standard deviations were done using commercial software (GraphPad Prism version 4.00 for Windows, GraphPad Software, San Diego California USA).

## 3. Results

**Figure 2:**
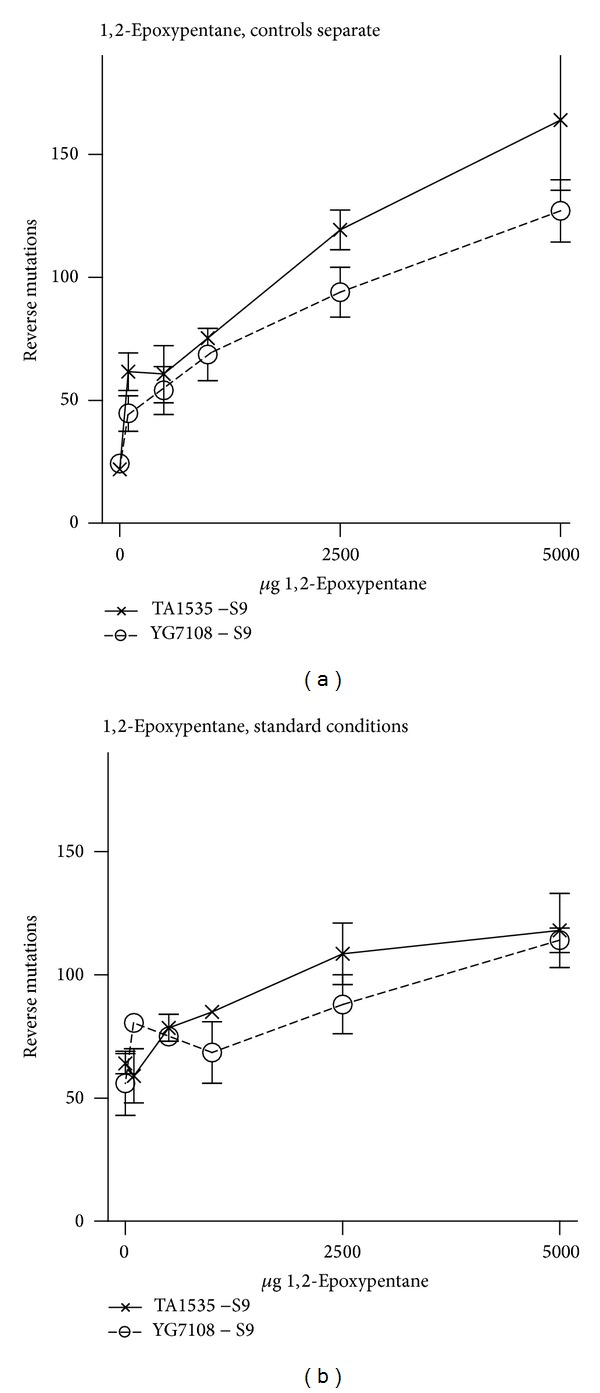
Bacterial mutagenicity of 1,2-epoxypentane in TA1535 and YG7108 without addition of S9-mix. With the aim to reduce the elevated base rate (standard conditions (b)), the control plates (0 *μ*g 1,2-epoxypentane) were placed in a separate incubator (a).

1,2-Epoxypentane was mutagenic in TA1535, YG7108 (Figures 2(a) and 2(b)), and TA100 (data not shown), exceeding the spontaneous mutation rate up to about 6-fold. No mutagenicity occurred in TA98 (data not shown). Notable differences of the response of 1,2-epoxypentane in TA1535 and YG7108 were not seen (Figures 2(a) and 2(b)). Remarkably the assay with 1,2-epoxypentane led to a strongly elevated spontaneous mutation rate (Figure 2(b), standard conditions). This was avoided by placing the control plates in a separate incubator (Figure 2(a)).

1-Pentene, 2-pentene,* cis*- and *trans*-2-pentene, and 3-methyl-1-butene were not mutagenic in any tester strain or under any test condition, summarized in Table 2. All tests with the amylenes were done with and without metabolic activation.

**Figure 3:**
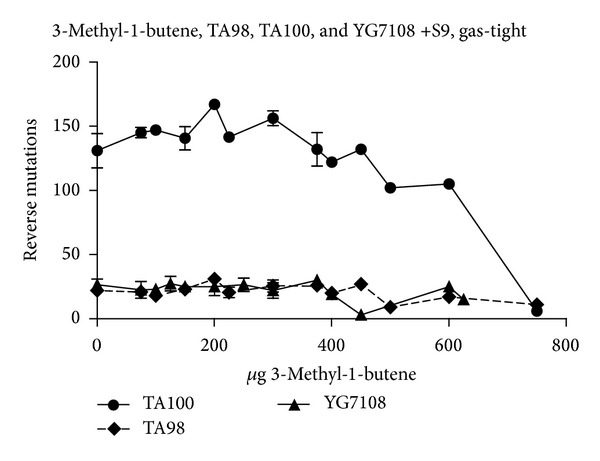
Results for 3-methyl-1-butene in TA98, TA100, and YG7108 with gas-tight preincubation and S9-mix. Three independent experiments were performed with differently spaced dose ranges.

Toxic effects occurred with and without addition of S9-mix, apparent by microcolony induction or reduced colony counts (latter is exemplarily shown in Figure 3). The racemate of 2-pentene revealed no toxicity in TA100 under standard conditions up to the highest tested amount of 5000 *μ*g/plate but according to the experiments using gas-tight preincubation the compound was toxic at and above 1000 *μ*g/plate. Accordingly toxicity occurred in YG7108 at and above 1500 *μ*g/plate upon gas-tight preincubation, whereas no toxicity was seen up to 2500 *μ*g/plate under standard conditions.

In case of microcolony induction more closely spaced test doses were applied with the aim to exclude a dose dependency (microcolonies can be recognized as toxic artifacts by a missing dose dependency). Single experiments showed in fact a tendency towards a dose trend. Such indications of dose trends could however not be confirmed by use of more closely spaced test doses. Therefore, in some experiments we applied an unusual number of different test concentrations (see Figure 3). If no toxicity occurred the compounds were tested up to 5000 *μ*g/plate.

## 4. Discussion

We observed no mutagenic effects of the short chained unsaturated hydrocarbons 1-pentene, 2-pentene, its stereo isomers *cis*-, *trans*-2-pentene, and 3-methyl-1-butene, even so we considered possible stereoselective effects in case of *cis*- and *trans*-2-pentene, possible evaporation of the compounds [8, 9], and bacterial repair [11]. YG7108 is sensitive for low molecular weight compounds which are false negative by use of the standard protocol and the parent strain TA1535. This was shown for compounds up to 4 C-atoms such as allyl chloride [10]. However, 1,2-epoxypentane exerted similar bacterial mutagenicity in YG7108 and TA1535. Possibly C-5 compounds mark the cutoff for the substrates of the deleted bacterial methyltransferases. In addition these experiments show that the oxirane which can be formed in the metabolism of 2-pentene would exert mutagenic effects in the bacterial reverse mutation test.

Data on mutagenic effects of 1,2-epoxypentane are only available from the summary of a study report which is cited by ECHA [4]. Mutagenicity in TA1535 was observed without S-9 mix from about 5000 *μ*g/plate (factor 2.3–2.6) onward and with metabolic activation from about 2500 *μ*g–5000 *μ*g/plate (factor 1.7–2.0) onward [4]. In our experiments 1,2-epoxypentane was mutagenic at and above 100 *μ*g per plate with a maximal increase of about 6-fold at 5000 *μ*g per plate. This discrepancy can be explained by evaporation of 1,2-epoxypentane into the incubator. In our experiments this led to exceptional high reverse mutations in the negative control plates and quite week mutagenic effects in the dosed samples. We attained a spontaneous reverse mutation rate within the historical range by placement of the control plates in a separate incubator (Figures 2(a) and 2(b)). Therefore, evaporation can lead to an underestimation of mutagenic effects of short chained oxiranes. This is in accordance with findings concerning ethylene oxide [3] and propylene oxide [3, 8, 16].

The question remains open why branched and un-saturated amylenes cannot be activated to mutagenic intermediates *in vitro*. Possibly the investigated amylenes are not efficiently epoxidized *in vitro* or oxidative metabolites which may occur are subjected to detoxification by the bacterial metabolism [17].

## 5. Conclusions

First, the investigated amylenes are not mutagenic, whereas the corresponding epoxide of 1-pentene (1,2-epoxipentane) exerted clear bacterial mutagenicity. Epoxidation of the investigated amylenes using rat liver S9-mix may not to be effective or is counterbalanced by detoxifying reactions. Second, weak bacterial mutagenicity of low molecular weight compounds such as 1,2-epoxipentane, ethylene-, or proplyene oxide can be caused by evaporation of compounds.

## Figures and Tables

**Table 1:** Reverse mutations for positive and negative controls.

Strain		TA98	TA100	YG7108
Solvent control	−S9	18 ± 6 (*N* = 16)	120 ± 17 (*N* = 17)	20 ± 6 (*N* = 18)
	+S9	20 ± 7 (*N* = 20)	145 ± 19 (*N* = 15)	26 ± 9 (*N* = 19)
Gas-tight	−S9	19 ± 7 (*N* = 10)	128 ± 18 (*N* = 9)	24 ± 9 (*N* = 13)
	+S9	21 ± 4 (*N* = 20)	141 ± 18 (*N* = 12)	23 ± 6 (*N* = 17)
Not gas-tight:				
3-NBA, 5 ng	−S9	709 ± 328 (*N* = 12)		
MMS, 200 *μ*g	−S9		1516 (*N* = 1)	805 ± 283 (*N* = 10)
MMS, 500 *μ*g	−S9		968 ± 175 (*N* = 13)	
MMS, 1000 *μ*g	−S9		1543 ± 40 (*N* = 2)	
2-AF	+S9	843 ± 228 (*N* = 19)	646 ± 117 (*N* = 13)	
2-AA, 5 *μ*g	+S9		781 ± 52 (*N* = 3)	
2-AA, 10 *μ*g	+S9	486 ± 175 (*N* = 3)		
NDEA, 500 *μ*g	+S9			225 ± 85 (*N* = 11)
NDEA, 1000 *μ*g	+S9			414 ± 360 (*N* = 16)

3-NBA: 3-nitrobenzanthrone; 2AF: 2-aminofluorene; 2-AA: 2-aminoanthracene; MMS: methyl methanesulfonate; NDEA: *N*-nitrosodimethylamine.

**Table 2:** Overview of all other results, not displayed as a figure.

Compound	Strain	Condition	Result
1-Pentene	TA98	±S9, gas-tight (*N* = 3) and not gas-tight (*N* = 7)	Negative
	TA100	±S9, gas-tight (*N* = 2) and not gas-tight (*N* = 7)	Negative
	YG7108	±S9, gas-tight (*N* = 5) and not gas-tight (*N* = 7)	Negative

2-Pentene (racemate)	TA98	±S9, gas-tight (*N* = 4) and not gas-tight (*N* = 4)	Negative
	TA100	±S9, gas-tight (*N* = 3) and not gas-tight (*N* = 4)	Negative
	YG7108	±S9, gas-tight (*N* = 3) and not gas-tight (*N* = 4)	Negative

*cis*-2-pentene	TA98	±S9, not gas-tight (*N* = 3)	Negative
	TA100	±S9, not gas-tight (*N* = 4)	Negative
	YG7108	±S9, not gas-tight (*N* = 3)	Negative

*trans-*2-pentene	TA98	±S9, not gas-tight (*N* = 3)	Negative
	TA100	±S9, not gas-tight (*N* = 4)	Negative
	YG7108	±S9, not gas-tight (*N* = 3)	Negative

3-Methyl-1-butene	TA98	−S9, not gas-tight (*N* = 2)	Negative
	TA100	−S9, not gas-tight (*N* = 2)	Negative
	YG7108	−S9, not gas-tight (*N* = 5)	Negative

## References

[1] HSDB http://toxnet.nlm.nih.gov/cgi-bin/sis/htmlgen?HSDB.

[2] Wistuba D, Nowotny HP, Träger O, Schurig V (1989). Cytochrome P-450-catalyzed asymmetric epoxidation of simple prochiral and chiral aliphatic alkenes: species dependence and effect of enzyme induction on enantioselective oxirane formation. *Chirality*.

[3] Canter DA, Zeiger E, Haworth S (1986). Comparative mutagenicity of aliphatic epoxides in *Salmonella*. *Mutation Research*.

[4] ECHA Substance identification. http://apps.echa.europa.eu/registered/data/dossiers/DISS-9eaef805-c5d0-3646-e044-00144f67d031/DISS-9eaef805-c5d0-3646-e044-00144f67d031_DISS-9eaef805-c5d0-3646-e044-00144f67d031.html#AGGR-a41a0eff-9de0-4379-bd0d-637cc33ac98d.

[5] ECHA Information on chemicals. http://www.echa.europa.eu/web/guest/information-on-chemicals.

[6] Gervasi PG, Longo V (1990). Metabolism and mutagenicity of isoprene. *Environmental Health Perspectives*.

[7] de Meester C, Mercier M, Poncelet F (1982). Non-mutagenicity of 2-methyl-2,3-epoxybutane and factors influencing the mutagenicity of 2,3-epoxybutane. *Journal of Applied Toxicology*.

[8] Westphal GA, Blaszkewicz M, Leutbecher M, Muller A, Hallier E, Bolt HM (1994). Bacterial mutagenicity of 2-chloro-1,3-butadiene (chloroprene) caused by decomposition products. *Archives of Toxicology*.

[9] Westphal GA, Bünger J, Schulz TG, Müller MM, Hallier E (2000). Mutagenicity of N-nitrosodiethylamine in the Ames test with *S. typhimurium* TA1535 is due to volatile metabolites and is not dependent on cytochrome P4502E1 induction. *Archives of Toxicology*.

[10] Emmert B, Bünger J, Keuch K (2006). Mutagenicity of cytochrome P450 2E1 substrates in the Ames test with the metabolic competent *S. typhimurium* strain YG7108pin3ERb_5_. *Toxicology*.

[11] Yamada M, Sedgwick B, Sofuni T, Nohmi T (1995). Construction and characterization of mutants of *Salmonella typhimurium* deficient in DNA repair of O^6^-methylguanine. *Journal of Bacteriology*.

[12] Castelain P, Criado B, Cornet M, Laib R, Rogiers V, Kirsch-Volders M (1993). Comparative mutagenicity of structurally related aliphatic epoxides in a modified *Salmonella*/microsome assay. *Mutagenesis*.

[13] Bellucci G, Lippi A, Marioni F (1984). Structure activity relationship of epoxides: different mutagenicity of the two diastereoisomeric 3-bromo-1,2-epoxycyclohexanes. *Chemico-Biological Interactions*.

[14] Maron DM, Ames BN (1983). Revised methods for the *Salmonella* mutagenicity test. *Mutation Research*.

[15] Mortelmans K, Zeiger E (2000). The Ames *Salmonella*/microsome mutagenicity assay. *Mutation Research*.

[16] Bootman J, Lodge DC, Whalley HE (1979). Mutagenic activity of propylene oxide in bacterial and mammalian systems. *Mutation Research*.

[17] Ensign SA, Allen JR (2003). Aliphatic epoxide carboxylation. *Annual Review of Biochemistry*.

